# Potential determinants of health-care professionals’ use of survivorship care plans: a qualitative study using the theoretical domains framework

**DOI:** 10.1186/s13012-014-0167-z

**Published:** 2014-11-15

**Authors:** Sarah A Birken, Justin Presseau, Shellie D Ellis, Adrian A Gerstel, Deborah K Mayer

**Affiliations:** Department of Health Policy and Management, Gillings School of Global Public Health, The University of North Carolina at Chapel Hill, 1103E McGavran-Greenberg, 135 Dauer Drive, Campus Box 7411, Chapel Hill, 27599-7411 NC USA; Institute of Health and Society, Newcastle University, Baddiley-Clark Building, Richardson Road, Newcastle upon Tyne, NE2 4AX UK; Department of Health Policy and Management, University of Kansas School of Medicine, Mail Stop 3044, 3901 Rainbow Boulevard, Kansas City, 66160, KS USA; School of Nursing, University of North Carolina at Chapel Hill, 2800 Carrington Hall CB# 7460, Chapel Hill, 27599 NC USA

**Keywords:** Cancer, Health-care professional, Survivorship care plan, Implementation, Theoretical domains framework

## Abstract

**Background:**

Survivorship care plans are intended to improve coordination of care for the nearly 14 million cancer survivors in the United States. Evidence suggests that survivorship care plans (SCPs) have positive outcomes for survivors, health-care professionals, and cancer programs, and several high-profile organizations now recommend SCP use. Nevertheless, SCP use remains limited among health-care professionals in United States cancer programs. Knowledge of barriers to SCP use is limited in part because extant studies have used anecdotal evidence to identify determinants. This study uses the theoretical domains framework to identify relevant constructs that are potential determinants of SCP use among United States health-care professionals.

**Methods:**

We conducted semi-structured interviews to assess the relevance of 12 theoretical domains in predicting SCP use among 13 health-care professionals in 7 cancer programs throughout the United States with diverse characteristics. Relevant theoretical domains were identified through thematic coding of interview transcripts, identification of specific beliefs within coded text units, and mapping of specific beliefs onto theoretical constructs.

**Results:**

We found the following theoretical domains (based on specific beliefs) to be potential determinants of SCP use: health-care professionals’ beliefs about the consequences of SCP use (benefit to survivors, health-care professionals, and the system as a whole); motivation and goals regarding SCP use (advocating SCP use; extent to which using SCPs competed for health-care professionals’ time); environmental context and resources (whether SCPs were delivered at a dedicated visit and whether a system, information technology, and funding facilitated SCP use); and social influences (whether using SCPs is an organizational priority, influential people support SCP use, and people who could assist with SCP use buy into using SCPs). Specific beliefs mapped onto the following psychological constructs: outcome expectancies, intrinsic motivation, goal priority, resources, leadership, and team working.

**Conclusions:**

Previous studies have explored a limited range of determinants of SCP use. Our findings suggest a more comprehensive list of potential determinants that could be leveraged to promote SCP use. These results are particularly timely as cancer programs face impending SCP use requirements. Future work should develop instruments to measure the potential determinants and assess their relative influence on SCP use.

**Electronic supplementary material:**

The online version of this article (doi:10.1186/s13012-014-0167-z) contains supplementary material, which is available to authorized users.

## Background

Survivorship care plans (SCPs)—written documents that ideally include a cancer treatment summary and plan for surveillance and preventive care—are intended to improve care communication and coordination, improve patient satisfaction, and reduce the burden of late effects in the nearly 14 million cancer survivors in the United States [1]. Evidence suggests that SCPs improve survivors’ satisfaction with care [2]; communication with providers [3],[4]; knowledge about cancer, treatment, and follow-up care [5]-[7]; peace of mind [8]; and engagement in healthy behaviors [9]. SCPs have also been found to improve cancer screening rates [10], decrease patient wait times, and increase cancer programs’ capacity for treating cancer patients by shifting survivors to follow-up care providers [4]. Over the last decade, the Institute of Medicine (IOM), the Commission on Cancer, and several other high-profile organizations have recommended that SCPs be used (i.e., developed and delivered to survivors and their primary care providers). Nevertheless, SCP use in US cancer programs remains limited [11]-[14].

Some cancer care quality improvement organizations have developed strategies to promote SCP use [15]. The effectiveness of these strategies may be limited by insufficient evidence regarding determinants of SCP use; most extant studies rely on anecdotal evidence as a basis for identifying determinants, typically focusing on the availability of time, reimbursement, and information technology for SCP use [11]-[13],[16],[17]. Theory-driven studies may contribute to more comprehensive understanding of SCP use. The objective of this study was to use theory to identify potential determinants of SCP use. Results may be used to inform theory-driven survey instruments to assess the relevance of the potential determinants in diverse populations and settings and, subsequently, strategies to promote SCP use. The need for evidence-based strategies mounts as SCP use requirements go into effect, such as Commission on Cancer program standards for SCP use, which go into effect in 2015 [18].

## Methods

### Participant identification

Clinical teams were identified in 36 cancer programs throughout the US with a wide range of annual incident cancers, program types, and cancer care quality improvement organization memberships. In each of these cancer programs, an employee responded to a survey of survivorship care plan use in a previous study [11], indicated that SCPs were used at the time of the survey (either “sometimes” or “regularly”), and consented to future contact. Three of these respondents submitted their surveys anonymously and were therefore excluded from the study, resulting in a sampling frame of 33 cancer programs. Of these, we selected ten that maximized variation in key cancer program characteristics (e.g., location, annual caseload).

### Recruitment

Our goal was to recruit all members of all clinical teams that used SCPs in each sampled cancer program; clinical teams that did not use SCPs were excluded from the study. To identify clinical teams, we emailed survey respondents to introduce them to the study. We then called survey respondents to obtain contact information for health-care professionals who used SCPs. When a survey respondent declined to participate, we requested contact information for another health-care professional within the cancer program who used SCPs. Once a health-care professional consented to participate, a snowball approach was used to identify the health-care professional’s team members (e.g., nurse to compile treatment summary, nurse practitioner to deliver SCP to survivor, see Additional file 1 for the participant identification protocol). When a health-care professional declined to participate and another health-care professional who used SCPs could not be identified, the cancer program was replaced with another cancer program with similar characteristics. Clinical teams consisted of as few as one member and as many as seven (mean = 2.2 clinical team members per cancer program; one cancer program had two clinical teams; six cancer programs had one clinical team that used SCPs), for a total of 13 health-care professional interviews in seven cancer programs.

## Materials

SCP use is a behavior that is likely to be influenced by determinants at organizational, group, and individual levels. To investigate potential determinants of SCP use across these levels, we selected the theoretical domains framework (TDF), a comprehensive framework of determinants of health-care professional behavior that synthesizes 33 psychological theories (e.g., theory of planned behavior [19], social cognitive theory [20]) into 12 domains based on a consensus exercise among experts in the field [21]. The TDF has been used to understand a variety of clinical behaviors including blood transfusion [22], prescription errors [23], and hand washing [24]. Tested strategies have been developed to leverage findings from TDF-based studies for designing behavior change interventions [25].

We developed a semi-structured interview guide (see Additional file 2) to elicit information regarding TDF domains (see detailed definitions in Additional file 3): knowledge about SCPs; skills using SCPs; whether using SCPs is aligned with health-care professionals’ social/professional role and identity; beliefs about capabilities to use SCPs; beliefs about consequences of using SCPs; whether health-care professionals are motivated to use SCPs; memory, attention, and decision processes around using SCPs; environmental context and resources for SCP use; social influences on SCP use; emotion about using SCPs; health-care professionals’ ability to regulate their SCP use; and the nature of SCP use [21]. The interview guide included between one and four questions per theoretical domain (a crosswalk between TDF domains and interview questions can be found in Additional file 4) Our definition of SCP use was based on the IOM’s framework, but we avoided leading participants to describe their SCP use as consistent with the IOM’s framework by asking them to describe their own SCP-related behavior (e.g., compiling treatment summaries); interviews focused on potential determinants of that behavior. Prompts were used to elicit thorough responses.

Two pilot interviews were conducted to ensure clarity and minimize interview length and repetitiveness. An investigator with expertise on the TDF reviewed pilot interviews to ensure adequate coverage and accurate representation of psychological constructs. The interview guide was then revised to incorporate missing elements.

### Procedure

The institutional review board at the University of North Carolina at Chapel Hill approved the study and waived written consent due to minimal risks to study participants. All interviews were conducted using the interview guide (Additional file 2). We obtained verbal consent from each participant. Interviews, including consent, were audio recorded and transcribed verbatim. An honorarium of $50 was offered for participation. Interviews lasted an average of 63 min (range: 37-86 min).

### Analysis

Interview transcript analysis involved identifying contextualized belief statements related to SCP use, categorizing statements into TDF domains and then into underlying theoretical constructs within domains, according to the following steps:

1. Coding interview transcripts at the TDF domain level
  We developed a coding manual (see Additional file 3) based on definitions from Michie et al. and Cane et al. [21],[26]. We coded any utterances that related to SCP use into domains. We used the coding manual for the initial indication of relevant TDF domain while being mindful of potential overlap across domains. To ensure consistency in coding, SB, SE, and RR (see Acknowledgements) independently coded one transcript, compared coding, and reconciled disagreements. In most cases, the reason for the discrepancy was obvious (e.g., misapplication of inclusion/exclusion criteria for a code, lapse of attention). Then, JP, who has expertise in the TDF, provided feedback to SB, SE, and RR on their coding. This process was repeated another two times until consensus was reached. SB, SE, and RR then independently coded the remaining interview transcripts.
2. Identifying context-specific beliefs
  SB and SE independently identified specific beliefs underlying statements within each coded domain (Table 1). A specific belief is a collection of responses from more than one interview participant with an underlying theme that suggested an influence on SCP use (e.g., “Gathering information is a barrier to using SCPs”) [27]. Specific beliefs on the same theme and opposites of a theme were coded as two instances of one specific belief. For example, utterances of “survivors benefit from SCPs,” “survivors receive better follow-up care because of SCPs,” and “I wonder whether SCPs actually benefit survivors” were all coded as *survivors benefit from SCPs*. SB and SE then compared coding and reconciled disagreements. This strategy was reviewed by JP to promote accurate content representation. Text units without underlying specific beliefs were excluded from further analysis. Using the method proposed by Francis et al., our initial analysis sample included ten participants in cancer programs with adequate diversity in cancer program characteristics to ensure theme saturation across specific beliefs [28]. Our stopping criterion was three additional interviews to ensure theme saturation. No additional specific beliefs emerged during analysis of the three additional interviews. As such, a total of 13 interviews were analyzed to identify specific beliefs.

**Table 1:** **Judgment of theoretical domain relevance** ^**a**^

Specific belief by domain	Total frequency of mentions	High frequency?^b^	Conflicting beliefs present?^c^	Strength of beliefs^d^
**Knowledge**				
*Using SCPs is required.*	*9*	*Yes*	*Yes*	*Moderate*
SCPs are a resource for survivors and their providers.	11	Yes	No	Weak
**Skills**				
Training facilitates SCP use.	3	No	No	Moderate
Using SCPs requires information technology skills.	7	No	No	Weak
*Using SCPs requires clinical knowledge.*	*11*	*Yes*	*No*	*Strong*
Using SCPs requires management skills.	2	No	No	Weak
Using SCPs requires communication skills.	8	Yes	No	Moderate
Using SCPs requires attention to detail.	2	No	No	Moderate
Using SCPs requires a clinical degree.	2	No	Yes	Weak
**Social/professional role and identity**				
*Using SCPs is compatible with my professional role.*	*13*	*Yes*	*Yes*	*Moderate*
**Beliefs about capabilities**				
SCPs need “just the right amount” of information to be useful to providers.	1	No	No	Moderate
SCP use depends on survivors’ characteristics.	10	Yes	No	Weak
A large volume of survivors is challenging to using SCPs.	5	No	No	Strong
I am confident in my ability to use SCPs.	10	Yes	no	Weak
*Gathering information is a barrier to using SCPs.*	*3*	*No*	*No*	*Strong*
***Beliefs about consequences***				
*The system as a whole benefits from SCP use.*	*13*	*Yes*	*Yes*	*Moderate*
*Providers benefit from using SCPs.*	*11*	*Yes*	*Yes*	*Moderate*
*Survivors benefit from SCPs.*	*13*	*Yes*	*Yes*	*Strong*
Survivors respond well to SCPs.	10	Yes	No	Weak
SCPs transition survivors from cancer treatment.	7	No	Yes	Weak
***Motivation and goals***				
*I advocate SCP use.*	*12*	*Yes*	*No*	*Strong*
*Using SCPs competes for my time.*	*12*	*Yes*	*Yes*	*Strong*
**Memory, attention, and decision processes**				
*A list helps me to use SCPs.*	*12*	*Yes*	*Yes*	*Weak*
When I forget to use SCPs, I use an old method of transitioning survivors.	2	No	No	Weak
I might not use SCPs if doing so were out of context	4	No	No	Weak
I schedule SCP use when it is most effective/efficient for me.	2	No	No	Weak
***Environmental context and resources***				
*SCPs are delivered at a visit devoted to transitioning survivors.*	*5*	*No*	*Yes*	*Strong*
*A “system” facilitates SCP use.*	*11*	*Yes*	*Yes*	*Strong*
*Information technology supports SCP use.*	*12*	*Yes*	*Yes*	*Strong*
*Funding facilitates SCP use.*	*9*	*Yes*	*Yes*	*Strong*
***Social influences***				
Survivor needs require SCP delivery to be as convenient as possible.	8	Yes	No	Moderate
*Using SCPs is an organizational priority.*	*4*	*No*	*Yes*	*Moderate*
*Influential people in the cancer program support SCP use.*	*12*	*Yes*	*Yes*	*Weak*
External stakeholders support SCP use.	6	No	Yes	Weak
SCP use varies across the cancer program.	6	No	Yes	Weak
*Using SCPs requires buy-in from people who could assist in using SCPs.*	*13*	*Yes*	*No*	*Strong*
**Emotion**				
It feels good to know that using SCPs helps survivors and their providers.	8	Yes	No	Moderate
Using SCPs helps me to feel calm when I transition survivors.	3	No	No	Weak
Using SCPs is stressful.	6	No	No	Moderate
**Behavioral regulation**				
Feedback facilitates SCP use.	11	Yes	No	Weak
Using SCPs takes practice.	6	No	No	Weak
I prepare to use SCPs by gathering information.	12	Yes	No	Weak
I “sell” SCP use to promote buy-in from others.	7	No	Yes	Moderate
I use SCPs as a tool for communicating with survivors.	12	Yes	No	Weak
*I modify how I use SCPs to meet specific needs.*	*10*	*Yes*	*Yes*	*Moderate*
I follow up with survivors to ensure that SCPs meet their needs.	3	No	No	Weak
**Nature of the behavior**				
Using SCPs is a “work in progress.”	6	No	Yes	Moderate
Survivors are expected to deliver SCPs.	1	No	No	Weak
SCPs should be kept up to date over time.	3	No	No	Weak
SCPs present privacy concerns.	1	No	Yes	Weak
There are many ways SCP use can be initiated.	4	No	No	Weak
*SCP use should begin during or prior to treatment.*	*5*	*No*	*Yes*	*Moderate*

^a^Domains and specific beliefs judged to be relevant are in italics.

^b^A relatively high frequency of specific beliefs (greater than the mean of seven participants referring to the specific belief).

^c^Conflicting beliefs across participants.

^d^Evidence of strong beliefs (i.e., participants indicated that the specific belief contributed or would contribute to SCP use).
3. Identifying relevant theoretical domains
  Specific beliefs were judged to be relevant based on the following criteria: (1) a relatively high frequency of specific beliefs (greater than the mean of seven participants referring to the specific belief), (2) conflicting beliefs across participants, and/or (3) evidence of strong beliefs (i.e., participants indicated that the specific belief contributed or would contribute to SCP use). All three criteria were considered concurrently to judge relevance. Consequently, specific beliefs with low frequency counts but greater respondent-perceived influence on SCP use were considered relevant. For example, the specific belief that “SCPs are delivered at a visit devoted to transitioning survivors” was deemed relevant despite low frequency because of participants’ strong beliefs about its influence on SCP use. DM, who is a practicing health-care professional and expert in survivorship care, helped interpret the importance of the specific beliefs from a clinical perspective. In some cases, clinical relevance outweighed the relevance designation algorithm described above. For example, “Using SCPs is a `work in progress’” and “Continuity of care is an important component of SCP use” have similar ratings; however, “Continuity of care is an important component of SCP use” was deemed highly relevant from a clinical perspective. Domains were judged to be relevant potential determinants of SCP use based on their number of relevant specific beliefs and their independently-judged clinical relevance.
4. Mapping constructs onto specific beliefs
  Using the method proposed by Francis et al., specific beliefs were analyzed to identify associated psychological constructs [27]. Once the relevant TDF domains were identified in step 3, SB and SE mapped the specific beliefs within those domains to representative theoretical constructs. For example, beliefs about the extent to which survivors and providers benefit from SCP use represent the psychological construct *outcome expectancies.* JP resolved discrepancies between the first two investigators.

## Results

### Cancer program and health-care professional characteristics

The seven cancer programs in the sampling frame represented each of the four Census Bureau regions of the United States (Northeast, Midwest, South, and West). Annual caseload ranged from 16-13,000 patients. The following program types were represented in the sample: community hospital cancer program, community hospital comprehensive cancer program, National Cancer Institute-designated comprehensive cancer program, pediatric cancer program, and teaching hospital cancer program. Participants’ cancer programs were members of the Association of Community Cancer Centers, American Hospital Association, Quality Oncology Practice Initiative, Commission on Cancer, and/or National Comprehensive Cancer Network. Although some current SCP use was required for inclusion in the study sample, participants represented cancer programs with a wide range of SCP use. In some cancer programs, SCP use was restricted to nascent pilot programs; in other cancer programs, SCPs had been used for as many as 5 years. Most cancer programs offered SCPs to survivors in all tumor groups, but one restricted SCP use to breast cancer survivors and another was implementing SCPs one tumor group at a time. Several cancer programs relied on one nurse or nurse practitioner to use SCPs; others integrated SCPs into programs with several employees. Participants included four physicians, five nurses, three patient navigators, and one patient educator in seven cancer programs. Participants’ role in using SCPs varied: in general, physicians only delivered SCPs, whereas other health-care professionals developed and/or delivered SCPs.

### Domains and associated constructs judged to be relevant to SCP use

**Figure 1:**
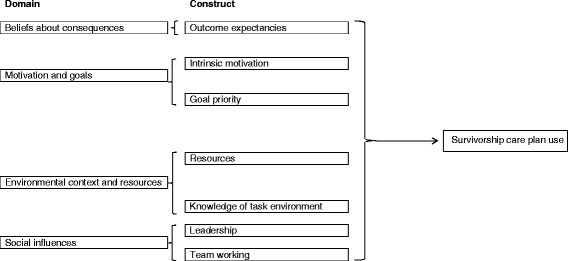
**Constructs representing determinants of survivorship care plan use by theoretical domain.**

Nineteen of 52 total specific beliefs were identified as most relevant to SCP use. Theoretical domains identified as most relevant to SCP use included the following: beliefs about consequences, motivation and goals, environmental context and resources, and social influences (relevant specific beliefs and domains are displayed in italics in Table 1; relevant domains are listed in the left column of Figure 1; and the six relevant constructs within relevant domains appear in the middle column of Figure 1.) We found that *outcome expectancies* were relevant to health-care professionals’ SCP use. Outcome expectancies are a construct within the beliefs about consequences domain that refers to assumptions regarding cognitive, emotional, behavioral, and affective outcomes associated with a behavior. Specifically, we found that SCP use depended upon health-care professionals’ assumptions about the extent to which SCPs would benefit survivors and health-care professionals: some participants were convinced that SCPs would help survivors to transition from treatment to follow-up care; other participants expressed concerns regarding the extent to which survivors used SCPs.

Within the *motivation and goals* domain, we found that *goal priority*—the order of importance or urgency that is placed on engaging in a behavior—was particularly relevant. Participants widely varied in the extent to which SCP use competed for their time with other job priorities. Participants whose time was devoted to SCP use were emphatic about the need for time devoted to SCP use, and those who split their time between using SCPs and other job priorities indicated that they had difficulty using SCPs. We also found that the construct *intrinsic motivation* within the motivation and goals domain was relevant to health-care professionals’ SCP use. Intrinsic motivation refers to engaging in a behavior for the sake of itself, as opposed to engaging in a behavior because doing so might result in an external benefit, such as financial incentives. One intrinsically motivated participant said, “I’m compelled to [use SCPs] because it’s my job. “[O]ne of the reasons I got hired was to do these treatment summaries and care plans or to get a plan in process that’s followed up and implemented, so…I’m driven to get it done.”

Participants consistently described the need for *resources*—a construct within the *environmental context and resources* domain that refers to the materials and staff used in enacting a behavior. One participant who enjoyed access to an able and willing staff and established processes for SCP use said, “The patient’s local providers are available through a program that [they] have been entered in, and so it’s in the record already, and we just find their fax number and enter it into the document, and it gets faxed to them.” Many other participants lamented the lack of referral systems for SCPs. Participants indicated that a visit devoted to SCP delivery and specific funding were needed for SCP use.

Frequent discussion related to the *social influences* domain widely varied. Some participants believed that SCP use could not continue without support from influential people, such as leaders. *Leadership* is a construct that refers to processes related to leading others, such as organizing, directing, coordinating, and motivating people toward achieving goals. Without explicit support from leaders, some health-care professionals were anxious about using SCPs. One participant described the anxiety she felt in the face of physician resistance to SCPs: “I hope I don’t get a call from that doctor telling me, `Why did you give my patient this information?’” Other participants indicated that they would be able to use SCPs even without support from influential people in their cancer program. In particular, we found that many participants relied on *team working*—collaborative effort to achieve a common goal—to use SCPs, with or without support from leaders. These health-care professionals indicated that SCP use primarily depended on buy-in from staff who could assist in using SCPs.

### Domains judged not to be relevant to SCP use

Eight of the 12 domains were considered not to be relevant to SCP use. These are displayed in regular font in Table 1. Participants were consistently *knowledgeable* about SCP use, believed they had the skills required, and varied little in their beliefs about the extent to SCP use was consistent with their *professional roles*. Few participants suggested that congruence between SCP use and their knowledge, skills, or professional role (or lack thereof) influences their SCP use.

*Beliefs about capabilities* were infrequently described and varied little: although participants indicated that using SCPs was challenging, they were confident in their ability to use SCPs.

Beliefs about *memory, attention, and decision processes* were weak and infrequently described. One participant said, “It is easy to get distracted, but then I will always go back and finish it up because that patient knows that I’m meeting with them at that one month follow-up [visit].”

Participants’ responses suggested that *emotions* including feeling good about helping survivors and health-care professionals and feeling stressed to develop SCPs did not influence SCP use. One participant said, “That’s where I feel more anxious. I sometimes don’t want to talk about that with patients, but I feel like it’s their right to know as a survivor so that they have the information.”

Regarding *behavioral regulation*, participants consistently reported that the methods they developed to facilitate SCP use did not strongly influence whether or not they used SCPs.

Specific beliefs related to the *nature of SCP use* were infrequently described. When participants did describe the nature of SCP use, they did not suggest that it influenced their SCP use. One participant said, “I think it’s considered a pretty basic document regarding a patient who’s been treated for cancer.”

## Discussion

SCP use is increasingly required of US cancer programs. For example, the Commission on Cancer will require SCP use as an indicator of cancer care quality beginning in 2015 [18]. In response to these requirements, many organizations have begun to develop strategies to promote SCP use [15]. Without empirical evidence of determinants of SCP use, these strategies may be unsuccessful. The few existing studies of SCP use have assessed a limited range of determinants [11],[13],[16],[17],[29]. By using a theory-based approach, our study uncovers a more comprehensive list of potential determinants.

Nevertheless, our study has several limitations. First, our study focused exclusively on potential determinants of SCP use among health-care professionals who reported at least some previous SCP use; our study did not compare health-care professionals who use SCPs to those who do not. Our goal in focusing on the circumstances under which health-care professionals who use SCPs sometimes elect not to do so was to ensure that participants could respond to each interview question; health-care professionals who have never used SCPs would be unable to answer questions regarding the nature of SCP use, for example. Second, based on the available demographic information that we had (gender, profession), participants did not systematically differ from health-care professionals who declined to participate; however, they may differ on unmeasured characteristics. Third, interview responses are subject to social desirability and attribution biases. Participants may have overstated their advocacy of SCP use and their attributions may not necessarily be reflective of actual determinants of SCP use. In particular, memory, attention, and decision processes may be difficult to self-report. Fourth, it is possible that the TDF does not include all potential determinants of SCP use; however, the TDF synthesizes 33 theories of behavior, so it is likely to be more comprehensive than existing studies of determinants of SCP use among health-care professionals or of studies selecting a single theory. Fifth, the generalizability of our findings may be limited. Although our study included participants from cancer programs with a wide range of characteristics, results are unlikely to be representative of all US cancer programs. Further limiting generalizability, the mean annual caseload in the sample was higher than in sampling frame (2,925 vs. 1,026, respectively). Health-care professionals in cancer programs with higher caseloads may have better infrastructure for SCP use or higher caseloads may place greater demands on the resources necessary to use SCPs. Limited generalizability may have contributed to premature saturation across specific beliefs. A more representative sample may have allowed us to identify new or different specific beliefs.

## Conclusions

Our study identifies six psychological constructs that are potential determinants of SCP use: the extent to which health-care professionals believe that SCP use will have positive outcomes for survivors; have access to resources required for SCP use; work in teams that support SCP use; and are intrinsically motivated to use SCPs, able to prioritize SCP use over competing demands, and led by people who prioritize SCP use. Few of these potential determinants have been assessed in empirical studies. Future studies should leverage our study results to assess the relative influence of each of the identified potential determinants. This may be accomplished by developing a survey instrument based on the results of this study. Such a survey could assess the extent to which potential determinants of SCP use identified in this study are generalizable to a nationally representative sample of health-care professionals in US cancer programs. Survey results would provide data for psychometric testing of the instrument and guidance for cancer programs implementing SCPs.

Our findings, in conjunction with the future studies proposed above, may be used to inform efforts to promote SCP use. The National Cancer Institute’s Community Cancer Centers Program, for example, offers technical assistance for SCP use and SCP templates. Quantitative studies are needed to assess the strength of the empirical relationships between the constructs that we identified and SCP use; however, our results suggest that the success of efforts to promote SCP use may be bolstered by attending to health-care professionals’ beliefs about the benefits of SCP use, devoting time to SCP use, ensuring that cancer program leaders champion SCP use, and creating teams that effectively collaborate to use SCPs. Our results also suggest that content such as didactic education and skills development may be unnecessary given sufficient health-care professional knowledge and capabilities in those who already use SCPs to some extent. Further, our results may provide insight into the discussion regarding electronic health records (EHR) as a means of facilitating SCP use. Conflicting beliefs regarding the extent to which information technology supports SCP use may represent varying levels of experience with EHR: health-care professionals without EHR may believe that information technology would facilitate SCP use, whereas those with EHR may have insight into the challenges associated with electronic SCPs. These insights are particularly timely as cancer programs face impending SCP use requirements, such as the Commission on Cancer’s, which go into effect in 2015 [18].

Our study also contributes to the growing body of literature that uses the TDF to identify health-care professionals’ use of health-care innovations. In particular, we use a rigorous analytical method that may offer guidance to other studies that apply the TDF. In April 2012, *Implementation Science* published a collection of articles related to the application of the TDF [30]. Empirical articles in the collection did not specify their criteria for judging domains’ relevance. In this study, we used an explicit process of judging frequency of specific beliefs, presence of conflicting beliefs across interviews, and evidence of strong beliefs (Table 1). This method promotes reproducibility of results. Future studies that apply the TDF should use similarly rigorous and explicit methods.

## Additional files

## Electronic supplementary material

Additional file 1: Participant identification protocol.(DOCX 14 KB)

Additional file 2: **Identifying barriers and facilitators to survivorship care plan use using the theoretical domains framework—** ***interview guide for semi structured interview.***(DOCX 21 KB)

Additional file 3: Codebook.(DOC 87 KB)

Additional file 4: Crosswalk between TDF domains and interview questions.(DOCX 22 KB)

Below are the links to the authors’ original submitted files for images.

Authors’ original file for figure 1

Authors’ original file for figure 2

## Abbreviations

EHR: electronic health record
IOM: Institute of Medicine
SCP: survivorship care plan
TDF: theoretical domains framework
