# Supplementary material for: Potential determinants of health-care professionals’ use of survivorship care plans: a qualitative study using the theoretical domains framework
Source: Implement Sci. 2014 Nov 15;9:167. doi: 10.1186/s13012-014-0167-z (PMC4236456; doi:10.1186/s13012-014-0167-z)
Supplement: Supplementary file 1 — Additional file 1: Participant identification protocol.(DOCX 14 KB) [file 13012_2014_167_MOESM1_ESM.docx]

**Additional file 1: Participant identification protocol**

The initial contact will be with the cancer program employee who completed the survey. Please ask the employee for the name of someone in the organization who uses SCPs (e.g., a medical oncologist who orders an SCP for a survivor; a nurse who compiles the treatment summary; a nurse practitioner who delivers the SCP to a survivor; front desk staff who delivers the SCP to a primary care provider; information technology staff who support the use of electronic SCPs; or and administrative staff who offer support for compiling or delivering the SCP). Please let the employee know that participating employees will receive a $50 Amazon.com gift card as thanks for their time.

Once a person who uses an SCP is identified, please obtain their contact information. The following guide is intended to be used with that person:

“Would it be OK with you if I record our call today? [If they ask why, say for research and training purposes.] Thank you for considering participating in this interview. If you agree to participate, you will be offered a $50 Amazon.com gift card to thank you for your time.

I would like to conduct a one-on-one interview with you. In addition, I’d like to conduct one-on-one interviews with anyone whom you work with on using SCPs. They would also be offered a $50 Amazon.com gift card.

“Several people may be involved in the use of SCPs. For example, a medical oncologist may order an SCP for a survivor; a nurse may compile the treatment summary; a nurse practitioner may deliver the SCP to a survivor; and front desk staff to deliver SCP to primary care provider. Sometimes information technology, staff support, the use of electronic SCPs, and administrative staff offer support for compiling or delivering the SCP.

“Would you please walk me through the life of an SCP, from when it’s ordered to when it’s delivered to a survivor and/or their primary care provider, and who is involved in each phase of the SCP’s life in your organization? Is there anyone in your organization who assists in the use of SCPs?”

[An employee is not eligible for inclusion in the study if the person being interviewed above has not ever personally interacted with the employee when using an SCP.

For eligible employees, please make sure to get their position title, specific name, and contact information. Then contact each one to set up an individual interview.

When you call the eligible employees, please confirm their involvement in SCP use. Remember, the key criterion is that they develop or deliver SCPs, or they personally interact with someone who does (e.g., perhaps they’re a medical oncologist who prescribes SCPs, or they’re an IT employee who supports clinicians in using electronic SCPs)]
