# Supplementary material for: Potential determinants of health-care professionals’ use of survivorship care plans: a qualitative study using the theoretical domains framework
Source: Implement Sci. 2014 Nov 15;9:167. doi: 10.1186/s13012-014-0167-z (PMC4236456; doi:10.1186/s13012-014-0167-z)
Supplement: Supplementary file 2 — Additional file 2: Identifying barriers and facilitators to survivorship care plan use using the theoretical domains framework— interview guide for semi structured interview.(DOCX 21 KB) [file 13012_2014_167_MOESM2_ESM.docx]

**Additional file 2: Identifying barriers and facilitators to survivorship care plan use using the theoretical domains framework – *Interview Guide for Semi Structured Interview***

**NOTE TO INTERVIEWER: Please use all items in prompts.**

60-75 minutes

**Introduction:**

Is this still a good time for you? Are you in a place where you can be free from distractions and feel free to give candid responses?

Would it be OK with you if I record this call? [If they ask why, say for research and training purposes.]

Thank you for agreeing to participate in this interview. The aim of the study is to help us understand more about the use of survivorship care plans (SCPs). SCPs are written documents that often, but not always, include the following information regarding care after cancer treatment is complete: treatment summary; surveillance plan; preventive care; and symptoms to report. Using SCPs sometimes involves several people in a cancer program. During this interview, I will ask about your perspective on using SCPs.

Your responses will help us understand how best to implement SCPs in practice. All of your responses will remain confidential and will only be reported in aggregate. You may choose to stop the interview at any time, and there is no penalty to your or your organization for not completing the interview. If you complete the interview, we will offer you a $50 Amazon.com gift card to thank you for your time.

Do you have any questions before we begin?

Let’s start by discussing what you know about SCPs in general.

1. What is the purpose of an SCP?
2. How are SCPs supposed to be used?
3. Is using SCPs required?

Prompts: By your organization? By external organizations (e.g., Commission on Cancer; National Comprehensive Cancer Network)? Anything else you can think of regarding SCPs in general?

Now let’s talk about doing what you do with respect to using SCPs.

1. Walk me through the steps you take in using SCPs.

Prompts: Does it depend? If so, what does it depend on? Does a post-treatment visit incite SCP use? Do you create SCPs? Do you compile treatment summaries? Do you deliver SCPs? Does someone ask you to use SCPs? Is an SCP handed off to you from anyone? Then what happens? When you’re done with an SCP, do you hand it off to anyone? Whom is the SCP delivered to, and how do they get it?

1. Which part or parts of this process that you just described do you see as central to what you do with respect to SCPs?

Prompts: E.g., delivering an SCP to a survivor in an appointment; compiling a treatment summary; faxing the SCP to a primary care provider

Please keep whatever you see as most central to what you do with respect to SCPs in mind for the rest of this interview. When thinking about your responses to my questions, please keep this in mind. So, when I say “using SCPs,” you should think about that/those central behavior[s] (e.g., developing, compiling, delivering to survivor, delivering to primary care provider, IT support, etc.).

1. From your perspective, what are the main barriers and facilitators to you using SCPs as you defined them above?

Now let’s talk about what happens before you use an SCP.

1. How do you prepare for using SCPs? Is there a method that you use to prepare to use SCPs?

Prompts: E.g., read up on recommended preventive care for survivors? Obtain template? Schedule appointment?

1. What are some of the skills that you use when using SCPs?

Prompts: What skills would a healthcare provider just beginning to use SCPs need to have or develop to effectively use SCPs? If you were to hire someone to do what you do, what sort of skills would they need? E.g., knowledge of pharmacology, information technology; research; communication

1. Do you feel as though you have the skills required to use SCPs?

Prompt: What additional skills training do you need? What additional skills training do others on your clinical team need?

1. How do you feel before you use SCPs?

Prompts: Do you ever feel any positive emotions? What about negative emotions? Do these emotions differ from when you first started using SCPs? Can you imagine how someone new at using SCPs might feel? E.g., empathetic, hopeful, positive, resentful, stressed, anxious, content, excited

1. In what ways do your feelings influence whether or how you use SCPs?

Prompts: E.g., if you feel anxious about using SCPs, are you less likely to use them?

Now let’s talk about what happens while you use an SCP.

1. What methods do you use for using SCPs?

Prompts: If-then plan, checklist, rules of thumb, reminders, prompts? Why? Why not?

1. What procedures or ways of working might encourage you to use SCPs?

Prompts: Audits, reminders, protocol or policy, or increasing monitoring of your behavior (e.g., as part of grant requirements)? Feedback on your SCP use? Incentives?

1. What difficulties do you encounter when using SCPs?

Prompts: information technology issues; social conflicts; problems with templates, sending SCPs, making appointments; pushback from staff or management; lack of reimbursement, support from managers, training, grant funding, reimbursement, staffing, own time; How do the difficulties you encounter when using SCPs now differ from the difficulties you encountered when you first started using SCPs? What kind of difficulties do you imagine someone new at using SCPs might encounter?

1. How confident are you in using SCPs despite these difficulties?
2. How confident are you that your clinical team can use SCPs?
3. How do you feel while you use SCPs?

Prompts: Do you ever feel any positive emotions? What about negative emotions? Do these emotions differ from when you first started using SCPs? Can you imagine how someone new at using SCPs might feel? E.g., empathetic, hopeful, positive, resentful, stressed, anxious, content, excited

1. Is there anything that helps you or makes it easier for you to use SCPs?

Prompts: What, specifically, helps you? E.g., compensated time, electronic health records, incentives. Which of these helps you most? Tell me how that works.

1. Is there anything that makes it difficult to use SCPs?

Prompts: What, specifically, makes it difficult? E.g., compensated time, electronic health records, incentives. Which of these makes it most difficult?

1. Does using SCPs ever slip your mind? If so, could you please give me an example?
2. Are there ever situations in which you get distracted from using SCPs? If so, could you please give me an example?

Prompts: E.g., an urgent patient situation arises; something more important comes up; a colleague interrupts you; thinking about next client; 6pm on a Friday and running late to pick up kids

1. How do you feel after you use SCPs?

Prompts: Do you ever feel any positive emotions? What about negative emotions? Do these emotions differ from when you first started using SCPs? Can you imagine how someone new at using SCPs might feel? E.g., empathetic, hopeful, positive, resentful, stressed, anxious, content, excited

Now I’ll ask a few general questions about using SCPs.

1. Have you ever used a method other than SCPs to transition survivors to a primary care provider? If so, how does using SCPs differ from what you did to transition survivors to a primary care provider previously?

Prompts: In terms of logistics. What needs to be done differently? Has it changed what you do or what others do?

1. What are the benefits of using SCPs?

Prompts: …to patients, colleagues, cancer program, self: promotion, cancer program accreditation, following guidelines, being respected by colleagues, etc.

1. What are the costs of using SCPs, financial and otherwise?

Prompts: To you, your cancer program, patients, primary care providers, etc.; e.g., time taken away from other tasks

1. Do you that feel the benefits of using SCPs outweigh the costs? How so?
2. How important is it to you to use SCPs?

Prompts: How much do you want to do it? Are you compelled to do it? Are there other tasks that you perform in your job that are more important? Why?

1. In what way is using SCPs compatible with other things that you do in your job?

Prompts: If so, how? Does using SCPs eliminate, minimize, or somehow facilitate your other duties? Or does using SCPs make it harder to perform your other duties?

Let’s talk about what people in your clinical team think about using SCPs.

1. In your opinion, how much does using SCPs align with what somebody in your position should be doing?

Prompts: E.g., Is meeting with survivors to discuss an SCP something that a nurse should be expected to do?

1. What influential individuals or groups are in favor of using SCPs? Please tell me about them and their perspective on using SCPs.

Prompts: e.g., clinical leaders, management, etc.

1. What influential individuals or groups are against using SCPs? Please tell me about them and their perspective on using SCPs.

Prompts: e.g., clinical leaders, management, etc.

1. Do you consider their opinions when using SCPs?

Prompts: Do their opinions cross your mind at all?

1. Do you think about the opinions of these influential people when considering whether or how you use SCPs?

Prompts: If you got the sense that others didn’t approve of SCPs, would that influence whether or how you use SCPs?

**Conclusion:**

That’s all the questions I have for you, has anything occurred to you about this topic that I haven’t asked about?
