# Supplementary material for: Potential determinants of health-care professionals’ use of survivorship care plans: a qualitative study using the theoretical domains framework
Source: Implement Sci. 2014 Nov 15;9:167. doi: 10.1186/s13012-014-0167-z (PMC4236456; doi:10.1186/s13012-014-0167-z)
Supplement: Supplementary file 3 — Additional file 3: Codebook.(DOC 87 KB) [file 13012_2014_167_MOESM3_ESM.doc]

**Additional file 3: Codebook**

NOTES:

1. Conceptual framework is the Theoretical Domains Framework (Michie et al., 2005)
2. Code domains, using constructs to justify the domain coding. See below for construct definitions.
3. Make sure to code question as well as response when response does not reflect content of question.
4. Code both affirmative and negative responses.
5. Code both straightforward identification of issues (e.g., I have sufficient skills to use SCPs) and discussion of the implications (e.g., I was able to make decisions quickly because I had the tools I needed to use SCPs).

| **Domain** | **Decision Rules** | **Examples** |
| --- | --- | --- |
| Knowledge | Appropriate use: context surrounding SCPs – what respondents know about Inappropriate use: How interviewees attempt to gain knowledge to inform their engagement in the behavior (this would be behavioral regulation) | “Oh, it’s **I view it more as a living document so that way when the patients have completed treatment and they are hopefully cancer free down the road five, ten, fifteen years, they’re able to take that document and give it to their primary care physician and move on with their daily living activities lives.** So let’s say if they’re a breast cancer patient and they had Herceptin and in maybe fifteen years they start developing cardiac issues, well they can take that treatment summary that they had and deliver it to the cardiologist and then that cardiologist can see what kind of chemotherapy they had and maybe that’s the reason why they’re having some cardiac issues or something along that line.” |
|
|
|
|
| Skills | Appropriate use: something helpful/necessary for behavior, directly related to behavior; something that one can acquire; an ability (vs. aptitude related to belief about capabilities) Inappropriate use: Abilities | “I think one of the best **skills I have is my past history of being a nurse** because you get used to talking – so the doctors would come in and they’d talk to the patients and they’d say these things and sometimes the doctors I think forget that they’re using medical terminology which sometimes people don’t know that means so then they kind of look at you and then when the doctor leaves then you just reiterate what they said and **you explain it down in terms to what they mean so that way they know what the terms mean** and what’s happening to them and everything. I think **having that medical terminology** as well as, yeah, the medical terminology I think has helped me so when I’m reading through, looking through their different side effects that they’re having, I know what that means and then I can put it in a treatment summary and don’t word it so medical but make it so the patients understand what it means.” |
|
|
|
|
|
|
| Social/professional role and identity | Appropriate use: evaluation of what someone in position would do/differentiation of who should be doing what; distinction between what the interviewee does/should be doing versus what someone else in the organization does/should be doing  Inappropriate use: | “**[Using SCPs is] one hundred percent aligned [with what someone in my position should be doing]**.”  “**I think of course I’m compelled to do it because it’s my job.** That’s like one of the reasons I got hired was to do these treatment summaries and care plans or to get a plan in process that’s followed up and implemented so for me **I’m driven to get it done** but at the same time, sometimes I think I lose track that there are other things I could be doing.” |
| Beliefs about capabilities | Appropriate use: respondent’s ability to do behavior/ sense of aptitude in the context in which they’re supposed to perform the behavior (vs. ability related to skills)  Inappropriate use: Beliefs about someone else’s capabilities | “[The electronic health record] makes [SCPs] **really easy to generate**.”  “**you start questioning yourself**. You’re like I’m pretty sure this is right, right? Sometimes it can be a little bit tricky because what they heard or what they interpreted the physician saying and what they’re actually saying to you like different whereas you know you’re pretty sure the physician did tell them X, Y, and Z. They just probably didn’t hear it because I think just processing all of that is so much to take in so **sometimes I do feel apprehension and like oh, I hope I told them the right information and I hope I don’t get a call from that doctor telling me why did you give my patient this information** but that’s one thing that I always do.” |
| Beliefs about consequences | Appropriate use: assumptions about what happens following engaging in the behavior Inappropriate use: | “I sometimes don’t want to talk about that with patients but I feel like it’s their right to know as a survivor so **that way they have the information** *[this is an assumption about the consequence of providing an SCP]* but there’s a fine line between giving them education and information but also **I don’t want to scare them**.” |
| Motivation and goals | Appropriate use: Inappropriate use: | “I go in early to do it and I check it everyday because **I do have to turn in numbers for my grant** you could say so the number of treatment summaries I’m doing and the number of new patients and encounters and follow ups and all that **so I am being held accountable to that and I’d like to keep my grant so that holds – and I guess in some ways that’s an incentive for me so that holds me really well to be accountable and to be motivated to get this done**.”  “I think of course I’m compelled to do it because it’s my job. That’s like one of the reasons I got hired was to do these treatment summaries and care plans or to get a plan in process that’s followed up and implemented so for me **I’m driven to get it done** but at the same time, sometimes I think I lose track that there are other things I could be doing.” |
| Memory, attention, and decision processes | Appropriate use: trigger for the respondent Inappropriate use: A trigger for someone other than the respondent | “I think it's easier to do the care plan, to perform the care plan at a timely visit, especially once you've seen the patient and are reviewing your own note. **I think it's more valuable to do it at that time, because everything's kind of fresh in your mind and you have everything you need at that moment to complete and be informed**. I think **it's much more difficult to try to do it afterward** because we see so many patients and to get it over with, just to get it over with, and to time it at the same time -- maybe that's a better expression -- to time it at the same time you're trying to sign out your own note makes it a closure, so that this way **you know you've completed what you need to do for the local physician as well as what you need to do for the patient's next visit**”  “**I’ve forgotten several patients to do it** because I’ve been – we have two locations and it’s just me right now so sometimes I have forgotten about the patients completely or if I’m on vacation. If you have a week off, you don’t know what’s coming up for that next week or there might be patients who are there that week finishing up so I may not reach all the patients just due to the way we have two sites and I may be at one site one day and I may miss a patient at the other site.”  “**It is easy to get distracted** but then I will always go back and finish it up because that patient knows that I’m meeting with them at that one month follow up so that holds me to be accountable to meet with that patient.” |
| Environmental context and resources | Appropriate use: resources for supporting behavior (e.g., training, EHR, staff); the context in which the interviewee does what s/he does with regard to the behavior  Inappropriate use: | “**EMR systems** do not self populate [the SCP] so an average is ninety minutes to fill out”  “**The patient's local providers are available** through a program that their local providers have been entered in, and so it's in the record already, and we just find their fax number and enter it into the document and it gets faxed to them.”  “There has always been a **survivorship program**.”  “None of our documentation is **reimbursable**.” |
| Social influences | Appropriate use: An interaction or perception of the respondent that influences the respondent’s engagement in the behavior  Inappropriate use: | “[Physicians] are fine with me meeting with the patients and everything but I think they forget and **it goes better if they’re able to explain to the patient who I am because it just creates more of a trustworthiness if the doctor says oh this is [?Kathleen 17:39?]. She’s one of the nurses here and she’s going to be doing this. I think they just forget about that so getting more physician buy in**.”  “**I hope I don’t get a call from that doctor telling me why did you give my patient this information**”  “That would mean then that **the nurses and the physicians would one, understand the importance of it** and two, are helping to produce them so I’m getting more – **I don’t like the word buy in but I’m getting more help from the different departments**” |
| Emotion | Appropriate use: how the person or someone they’re interacting with is feeling (patient), just for documentation in this case given that it was a popular interpretation of the question among respondents  Inappropriate use: | “That’s where **I feel more anxious**. I sometimes don’t want to talk about that with patients but I feel like it’s their right to know as a survivor so that way they have the information but there’s a fine line between giving them education and information but also I don’t want to scare them.”  “I guess you feel a **sense of accomplishment, that you'll be ready** to see the patient the next time with a really good sense of where, what happened, and that if somebody else picks this up that they would be able to know pretty much what happened with your interaction and where to go.” |
| Behavioral regulation | Appropriate use: respondents’ attempt to influence process related to the behavior or make the behavior “work” for them  Inappropriate use: the way things are done in the organization (this would be nature of the behavior) | “Well when I first meet with the patient on the first day of radiation oncology, we do a distress thermometer. I’m going blank on what kind we use. I just went blank on what it was called. Oh, the NCCN I think it is – **the distress thermometer and then I give them that and that clues me** into if they’re having any social issues like transportation troubles or insurance issues and then also **that really starts a great conversation** with them because if they mark yes or no, then you can ask and elaborate more so I kind of assess them at the very beginning through that and then also the doctor’s H&Ps will state if there is any social history and family history and things like that **so I already have a background information before I meet the patient**.” |
| Nature of the behavior | Appropriate use: process – what the interviewee does in relation to the behavior; what the typical process is in the organization (i.e., what anyone else in the same position in the organization would do)  Inappropriate use: What someone else does in relation to the behavior (this might be coded as environmental context.); how the interviewee in particular engages in the behavior (different from typical methods in the organization; this would be coded as behavioral regulation) | “**I think it's considered a pretty basic document regarding a patient who's been treated for cancer.** There's no particular benefit or, what's the word, merit. It's considered part of the job.”  “We’re such a mobile society that in the smaller towns, they go and get the scans done and the x-rays, the CT but then they come here to get treated and then they go somewhere else and **you don’t have access to those outside records**. If the doctors or the physicians or whatever outside records don’t come to you**, you have to try to figure out where that was from and go find it.**” |

**Definitions**

**Knowledge**

Knowledge

An awareness of the existence of something.

Schemas

A collection of basic knowledge about a concept or entity that serves as a guide to perception, interpretation, imagination or problem solving.

Mindsets

An established set of attitudes regarded as typical of a particular group's social or cultural values; the outlook, philosophy, or values of a person; frame of mind, attitude, disposition.

Illness representations

Organised beliefs of illness acquired through the media, personal experience and from family and friends' experiences, beliefs, descriptions and knowledge of particular disorders.

Procedural knowledge

Knowing how to do something.

**Skills**

Skills

An ability or proficiency acquired through training and/or practice.

Competence

One's repertoire of skills, and ability especially as it is applied to a task or set of tasks.

Ability

Competence or capacity to perform a physical or mental act. Ability may be either unlearned or acquired by education and practice.

Skill assessment

A judgment of the quality, worth, importance, level, or value of an ability or proficiency acquired through training and practice.

Practice/skills development

Repetition of an act, behaviour, or series of activities, often to improve performance or acquire a skill.

Interpersonal skills

An aptitude enabling a person to carry on effective relationships with others, such as an ability to cooperate, to assume appropriate social responsibilities or to exhibit adequate flexibility.

Coping strategies

An action, series of actions, or a thought process used in an attempt to reduce stress or used to modify one's reaction to a stressful/unpleasant situation. Coping strategies typically involve a conscious and direct approach to problems.

**Social/professional role and identity**

Identity

An individual's sense of self defined by a) a set of physical and psychological characteristics that is not wholly shared with any other person and b) a range of social and interpersonal affiliations (e.g., ethnicity) and social roles

Professional identity/boundaries/role

The characteristics by which an individual is recognised relating to, connected with or befitting a particular profession.

Social identity

The set of behavioural or personal characteristics by which an individual is recognizable [and portrays] as a member of a social group

Group norms

Any behaviour, belief, attitude or emotional reaction held to becorrect or acceptable by a given group in society.

Alienation

Estrangement from one's social group; a deep seated sense of dissatisfaction with one's personal experiences that can be a source of lack of trust in one's social or physical environment or in oneself; the experience of separation between thoughts and feelings.

Organisational commitment

A distinctive pattern of thought and behaviour shared by members of the same organisation and reflected in their language, values, attitudes, beliefs and customs.

**Beliefs about capabilities**

Self-efficacy

An individual's capacity to act effectively to bring about desired results, as perceived by the individual.

Control—of behaviour and material and social environment

Authority, power or influence over events, behaviours, situations or people. Authority, power or influence over events, behaviours, situations or people.

Perceived competence

An individual's belief in his or her ability to learn and execute skills.

Self-confidence

Self-assurance or trust in one's own abilities, capabilities and judgment.

Professional confidence

An individual's belief in his or her repertoire of skills, and ability especially as it is applied to a task or set of tasks.

Empowerment

The promotion of the skills, knowledge and confidence necessary to take great control of one's life as in certain educational or social schemes; the delegation of increased decision-making powers to individuals or groups in a society or organization

Self-esteem

The degree to which the qualities and characteristics contained in one's self- concept are perceived to be positive.

Perceived behavioural control

An individual's perception of the ease or difficulty of performing the behaviour of interest.

Optimism

The attitude that outcomes will be positive and that people's wishes or aims will ultimately be fulfilled

Pessimism

The attitude that things will go wrong and that people's wishes or aims are unlikely to be fulfilled

**Beliefs about consequences**

Outcome expectancies

Cognitive, emotional, behavioural, and affective outcomes that are assumed to be associated with future or intended behaviours. These assumed outcomes can either promote or inhibit future behaviours

Anticipated regret

A sense of the potential negative consequences of a decision that influences the choice made: for example an individual may decide not to make an investment because of the feelings associated with an imagined loss.

Appraisal

The cognitive evaluation of a phenomenon or event. In theories of emotions, cognitive appraisals are seen as determinants of emotional experience

Evaluation

A careful examination or overall appraisal of something to determine its worth, value or desirability; a determination of the success of something in achieving defined goals; the interpretation of test results and experimental data.

Review

To look over or through in order to correct or improve; to revise

Consequents

An outcome of behaviour in a given situation.

Attitudes

Any subjective belief or evaluation associated with an object or behaviour.

Contingencies

A conditional probabilistic relation between two events. Contingencies may be arranged via dependencies or they may emerge by accident.

Reinforcement

A process in which the frequency of a response is increased by a dependent relationship or contingency with a stimulus.

Punishment

The process in which the relationship between a response and some stimulus or circumstance results in the response becoming less probable; a painful, unwanted or undesired event or circumstance imposed as a penalty on a wrongdoer.

Incentives

An external stimulus, such as condition or object, that enhances or serves as a motive for behaviour

Rewards

Return or recompense made to, or received by a person contingent on some performance

Beliefs

The thing believed; the proposition or set of propositions held true.

Unrealistic optimism

Return or recompense made to, or received by a person contingent on some performance.

Salient events/ critical incidents

Occurrences that one judges to be distinctive, prominent or otherwise significant

Characteristics of outcome expectancies–physical, social, emotional

Characteristics of the cognitive, emotional and behavioural outcomes that individuals believe are associated with future or intended behaviours and that are believed to either promote or inhibit these behaviours. These include whether they are sanctions/rewards, proximal/distal, valued/not valued, probable/improbable, salient/not salient, perceived risks or threats

Sanctions

A punishment or other coercive measure, usually administered by a recognised authority, that is used to penalise and deter inappropriate or unauthorised actions

Rewards, proximal/ distal, valued/ not valued, probable/ improbable

Return or recompense made to, or received by a person contingent on some performance.

Salient events/critical incidents

Occurrences that one judges to be distinctive, prominent or otherwise significant

Threat

A condition that is appraised as a danger to oneself or well-being or to a group

**Motivation and goals**

Intention

A conscious decision to perform a behaviour; a resolve to act in a certain way or an impulse for purposeful action. In experiments, intention is often equated with goals defined by the task instruction

Stability of intention

Ability of one's resolve to remain in spite of disturbing influences

Certainty of intention

A conscious decision to perform a behaviour; a resolve to act in a certain way or an impulse for purposeful action. In experiments, intention is often equated with goals defined by the task instruction.

Goals (autonomous, controlled)

Assuredness of one's resolve to act in a certain way.

Goal target/setting

A process that establishes specific time based behaviour targets that are measurable, achievable and realistic

Goal priority

Order of importance or urgency of end states toward which one is striving.

Intrinsic motivation

An incentive to engage in a specific activity that derives from the activity itself rather than because of any external benefits that might be obtained.

Commitment

The act of binding yourself (intellectually or emotionally) to a course of action.

Distal and proximal goals

Desired state of affairs of a person or system, these may be closer (proximal) or further away (distal).

Transtheoretical model and stages of change

A model that proposes that behaviour change is accomplished through five specific stages: Pre-contemplation, Contemplation, Preparation, Action, and Maintenance

**Memory, attention, and decision processes**

Memory

The ability to retain information or a representation of a past experience, based on the mental processes of learning or encoding retention across some interval of time, and retrieval or reactivation of the memory; specific information of a specific past

Attention

A state of awareness in which the senses are focussed selectively on aspects of the environment and the central nervous system is in a state of readiness to respond to stimuli.

Attention control

The extent to which a person can concentrate on relevant cues and ignore all irrelevant cues in a given situation.

Decision making

The cognitive process of choosing between two or more alternatives, ranging from the relatively clear cut to the complex

**Environmental context and resources**

Resources/material resources (availability and management)

Commodities and human resources used in enacting a behavior

Environmental stressors

External factors in the environment that cause stress

Person-environment interaction

Interplay between the individual and their surroundings

Knowledge of task environment

Knowledge of the social and material context in which a task is undertaken

**Social influences**

Social support

The apperception or provision of assistance or comfort to others, typically in order to help them cope with a variety of biological, psychological and social stressors. Support may arise from any interpersonal relationship in an individual's social network, involving friends, neighbours, religious institutions, colleagues, caregivers or support groups.

Social/group norms

Any behaviour, belief, attitude or emotional reaction held to be correct or acceptable by a given group in society

Organisational development

The application of principles and practices drawn from psychology, sociology and related fields to the planned improvement of organisational effectiveness.1

Leadership

The processes involved in leading others, including organising, directing, coordinating and motivating their efforts toward achievement of certain group or organisation goals

Team working

Cooperative effort toward a common goal or on a common project

Group conformity

The act of consciously maintaining a certain degree of similarity to those in your general social circles

Organisational climate/culture

A distinctive pattern of thought and behaviour shared by members of the same organisation and reflected in their language, values, attitudes, beliefs and customs

Social pressure

The exertion of influence on a person or group by another person or group.

Power/hierarchy

The capacity to influence others, even when they try to resist this influence.

Professional boundaries

The bounds or limits relating to, or connected with a particular profession or calling.

Professional roles

The behaviour considered appropriate for a particular kind of work or social position.

Management commitment

The binding of a governing body of an organization or business to a course of action.

Supervision

Management by overseeing the performance or operation of a person of group

Inter-group conflict

Disagreement or confrontation between two or more groups and their members. This may involve physical violence, interpersonal discord, or psychological tension

Champions

To fight for another or for a cause

Social comparisons

The process by which people evaluate their attitudes, abilities, or performance relative to others

Group Identity

The set of behavioural or personal characteristics by which an individual is recognizable [and portrays] as a member of a group

Social identity

The set of behavioural or personal characteristics by which an individual is recognizable [and portrays] as a member of a social group

Organisational commitment

An employee's dedication to an organisation and wish to remain part of it. Organisational commitment is often described as having both an emotional or moral element and a more prudent element

Alienation

Estrangement from one's social group; a deep seated sense of dissatisfaction with one's personal experiences that can be a source of lack of trust in one's social or physical environment or in oneself; the experience of separation between thoughts and feelings

Feedback

The return of information about progress on or the outcome of a process or activity.

Conflict—competing demands, conflicting roles

The actual or perceived incompatibility between the performance of two or more behaviours

**Emotion**

Affect

An experience or feeling of emotion, ranging from suffering to elation, from the simplest to the most complex sensations of feelings, and from the most normal to the most pathological emotional reactions

Stress

A state of physiological or psychological response to internal or external stressors

Anticipated regret

A sense of the potential negative consequences of a decision that influences the choice made: for example an individual may decide not to make an investment because of the feelings associated with an imagined loss

Fear

An intense emotion aroused by the detection of imminent threat, involving an immediate alarm reaction that mobilises the organism by triggering a set of physiological changes

Burn-out

Physical, emotional or mental exhaustion, especially in one's job or career, accompanied by decreased motivation, lowered performance and negative attitudes towards oneself and others

Cognitive overload/tiredness

The situation in which the demands placed on a person by mental work are greater than a person's mental abilities

Threat

A condition that is appraised as a danger to oneself or well-being or to a group

Positive/negative affect

The internal feeling/state that occurs when a goal has/has not been attained, a source of threat has/has not been avoided, or the individual is/is not satisfied with the present state of affairs

Anxiety

A mood state characterised by apprehension and somatic symptoms of tension in which an individual anticipates impending danger, catastrophe or misfortune

Depression

A mental state that presents with depressed mood, loss of interest or pleasure, feelings of guilt or low self-worth, disturbed sleep or appetite, low energy, and poor concentration

Implementation intention

The plan that one creates in advance of when, where and how one will enact a behavior

**Behavioral regulation**

Action planning

The action or process of forming a plan regarding a thing to be done or a deed

Self-monitoring

A method used in behavioural management in which individuals keep a record of their behaviour, especially in connection with efforts to change or regulate the self; a personality trait reflecting an ability to modify one's behaviour in response to situation

Goal priority

Order of importance or urgency of end states toward which one is striving

Generating alternatives

Thinking of other ways of dealing with a situation or problem

**Nature of the behavior**

Feedback

The return of information about progress on or the outcome of a process or activity

Moderators of intention-behaviour gap

Factors that affect the relationship between what one intends to do and what one actually does

Project management

Defining and achieving targets while optimizing the use of resources over the course of a project

Barriers and facilitators

In psychological contexts barriers/facilitators are mental, emotional or behavioural limitations/strengths in individuals or groups

Routine/automatic/habit

A well-learned behaviour or automatic sequence of behaviours that is relatively situation specific. At its extreme the behavior has become a reflex; independent of motivation, cognitive influence or conscious control

Breaking habit

To discontinue a behaviour or sequence of behaviours that is automatically activated by relevant situational cues

Direct experience

The experience gained through immediate sense perception.

Past behavior

Previous manner of conducting oneself.

Representation of tasks

A mental model of goal-directed activities

Stages of change model

A model that proposes that behaviour change is accomplished through five specific stages: Pre-contemplation, Contemplation, Preparation, Action, and Maintenance
