# Supplementary material for: Potential determinants of health-care professionals’ use of survivorship care plans: a qualitative study using the theoretical domains framework
Source: Implement Sci. 2014 Nov 15;9:167. doi: 10.1186/s13012-014-0167-z (PMC4236456; doi:10.1186/s13012-014-0167-z)
Supplement: Supplementary file 4 — Additional file 4: Crosswalk between TDF domains and interview questions.(DOCX 22 KB) [file 13012_2014_167_MOESM4_ESM.docx]

**Additional file 4. Crosswalk between TDF Domains and Interview Questions**

| **Domains*** | **Items** |
| --- | --- |
|  |  |
| **Knowledge** |  |
| Do they know about the guideline? | Would you please describe what you know about SCPs?  Prompts (make sure all are addressed in response):   - What are they? - Why are they supposed to be used? - How are they supposed to be used? - For whom are they supposed to be used? - When are they supposed to be used? |
| What do they think the guideline says? | Would you please describe what you know about SCPs?  Prompts (make sure all are addressed in response):   - What are they? - Why are they supposed to be used? - How are they supposed to be used? - For whom are they supposed to be used? - When are they supposed to be used? |
| What do they think the evidence is? | Would you please describe what you know about SCPs?  Prompts (make sure all are addressed in response):   - What are they? - Why are they supposed to be used? - How are they supposed to be used? - For whom are they supposed to be used? - When are they supposed to be used? |
| Do they know they should be doing x? | Would you please describe SCP use practices in your cancer program?  Prompts (make sure all are addressed in response):   - Why are they being used in your cancer program (i.e., compliance with professional society requirements)? - How are they supposed to be used? - How/by whom is SCP use initiated? What happens subsequently? - Where do SCPs end up? With whom? - For whom are they supposed to be used? - When are they supposed to be used? - For which tumor groups are SCPs supposed to be used? |
| Do they know why they should be doing x? | Would you please describe SCP use practices in your cancer program?  Prompts (make sure all are addressed in response):   - Why are they being used in your cancer program (i.e., compliance with professional society requirements)? - How are they supposed to be used? - How/by whom is SCP use initiated? What happens subsequently? - Where do SCPs end up? With whom? - For whom are they supposed to be used? - When are they supposed to be used? - For which tumor groups are SCPs supposed to be used? |
| **Skills** |  |
| Do they know how to do x? | - Walk me through the steps required to use an SCP. What skills are required? - Do you feel that you have the skills required to use SCPs? What additional skills training might you need? |
| How easy or difficult do they find performing x to the required standard in the required context? | Is using SCPs more difficult or easier than using another method of enhancing outcomes for individuals with cancer following completion of acute cancer treatment (e.g., survivorship clinic)? In what ways is using SCPs more or less difficult than any other method? |
| **Social/professional role and identity** |  |
| What is the purpose of the guidelines? | [No related question.] |
| What do they think about the credibility of the source? | Tell me about how you feel about SCPs as a means of improving survivors’ outcomes. |
| Do they think guidelines should determine their behaviour? | How do you feel about SCPs as an exclusive method of improving survivors’ outcomes? |
| Is doing x compatible or in conflict with professional standards/identity? (prompts: moral/ethical issues, limits to autonomy) | Please tell me about how SCPs relate to your sense of your role as a provider. |
| Would this be true for all professional groups involved? | [No related question.] |
| **Beliefs about capabilities** |  |
| How difficult or easy is it for them to do x? (prompt re. internal and external capabilities/constraints) | How easy or difficult is it to use SCPs? |
| What problems have they encountered? | What problems/difficulties do you encounter in using SCPs? |
| What would help them? | What might help you to overcome these problems/difficulties? |
| How confident are they that they can do x despite the difficulties? | How confident are you about using SCPs, despite these difficulties? |
| How capable are they of maintaining x? | How confident are you that you could continue to use SCPs? |
| How well equipped/comfortable do they feel to do x? | - How comfortable are you using SCPs? - How prepared do you feel to use SCPs? |
| **Beliefs about consequences** |  |
| What do they think will happen if they do x? (prompt re themselves, patients, colleagues and the organisation; positive and negative, short term and long term consequences) | - What do you perceive to be the benefits of using SCPs? (Prompts: To survivors, oncology providers, primary care providers, the cancer program… positive and negative, long-term and short-term) - What are the effects of your use of SCPs are on patients? - What are the effects of your use of SCPs are on you? - What are the effects of your use of SCPs are on your cancer program? |
| What are the costs of x and what are the costs of the consequences of x? | What do you perceive to be the disadvantages of using SCPs? |
| What do they think will happen if they do not do x? (prompts) | What would happen if you didn’t use SCPs? |
| Do benefits of doing x outweigh the costs? | Do the advantages of SCP use outweigh the disadvantages? Why? |
| How will they feel if they do/don’t do x? (prompts) | How would you feel if you didn’t use SCPs? |
| Does the evidence suggest that doing x is a good thing? | [No related question. Covered in knowledge domain.] |
| **Motivation and goals** |  |
| How much do they want to do x? | How much do you want to use SCPs? |
| How much do they feel they need to do x? | How much do you feel that you need to use SCPs? |
| Are there other things they want to do or achieve that might interfere with x? | Might using SCPs prevent you from or make it difficult to achieve any of your other objectives? |
| Does the guideline conflict with others? | Is using SCPs incompatible with other efforts that you make to enhance survivors’ outcomes? |
| Are there incentives to do x? | Are any incentives offered for SCP use? |
| **Memory, attention, and decision processes** |  |
| Is x something they usually do? | - Walk me through your thought processes when you are deciding whether or not to use SCPs. - Is using SCPs your standard method of enhancing outcomes for individuals with cancer following completion of acute cancer treatment? |
| Will they think to do x? | - Walk me through your thought processes when you are deciding whether or not to use SCPs. - Does using SCP ever slip your mind? If so, please describe a situation in which using an SCP slipped your mind? |
| How much attention will they have to pay to do x? | - Walk me through your thought processes when you are deciding whether or not to use SCPs. - Is using SCP something that you automatically do? If so, please describe the steps that you go through in using an SCP (i.e., what makes it automatic? Are there physical prompts, for example?). If not, when does your discretion come into play (e.g., are there decisions that must be made that determine whether SCPs will be used? For example, templates are sometimes available but not always)? - Does the decision regarding whether or not to use an SCP require a lot of thought? |
| Will they remember to do x? How? | - Walk me through your thought processes when you are deciding whether or not to use SCPs. - Does using SCP ever slip your mind? If so, please describe a situation in which using an SCP slipped your mind? |
| Might they decide not to do x? Why? (prompt: competing tasks, time constraints) | - Walk me through your thought processes when you are deciding whether or not to use SCPs. - Under what conditions might you decide not to use SCPs? |
| **Environmental context and resources** |  |
| To what extent do physical or resource factors facilitate or hinder x? | - Which resources help you to use SCPs? - Lack of which resources makes it difficult to use SCPs? |
| Are there competing tasks and time constraints? | Are there any competing tasks or time constraints that might influence whether you use SCPs or not? |
| Are the necessary resources available to those expected to undertake x? | To what extent do you have the necessary resources to use SCP? |
| **Social influences** |  |
| To what extent do social influences facilitate or hinder x? (prompts: peers, Social/group norms managers, other professional groups, patients, relatives) | - Is there any individual, group, or organization that approves of you using SCPs? How important is it to you that those individuals, groups, or organizations approve of your SCP use? - Is there any individual, group, or organization that disapproves of you using SCPs? How important is it to you whether those individuals, groups, or organizations disapprove of your SCP use? - In what ways do the views of others affect your SCP use? |
| Will they observe others doing x (i.e. have role models)? | - Is there consensus in the profession about how to approach enhancing outcomes for individuals with cancer following completion of acute cancer treatment? - To what extent do your colleagues use SCPs? |
| **Emotion** |  |
| Does doing x evoke an emotional response? If so, what? | How do you feel when you use SCPs? |
| To what extent do emotional factors facilitate or hinder x? | Do your feelings when using SCPs influence whether or how you use SCPs? If so, how? |
| How does emotion affect x? | Do your feelings when using SCPs influence the SCPs themselves? |
| **Behavioral regulation** |  |
| What preparatory steps are needed to do x? (prompt re individual and organizational) | What do you need to do to use an SCP? |
| Are there procedures or ways of working that encourage x? | Are there procedures or ways of working that might encourage you to use SCPs? |
| **Nature of the behavior** |  |
| What is the proposed behaviour (x)? | Please describe what it means to use an SCP. |
| Who needs to do what differently when, where, how, how often and with whom? | How does using SCPs differ from the method you use to use to enhance survivors’ outcomes? |
| How do they know whether the behaviour has happened? | [No related question.] |
| What do they currently do? | How does using SCPs differ from the method you use to use to enhance survivors’ outcomes? |
| Is this a new behaviour or an existing behaviour that needs to become a habit? | How does using SCPs differ from the method you use to use to enhance survivors’ outcomes? |
| Can the context be used to prompt the new behaviour? (prompts: layout, reminders, equipment) | Are there ways that your organization could make your SCP use more consistent? |
| How long are changes going to take? | [No related question.] |
| Are there systems for maintaining long term change? | Are there efforts/initiatives to make SCP use sustainable for you? |

*Domain questions listed in Michie S, Johnston M, Abraham C, Lawton R, Parker D, Walker A: Making psychological theory useful for implementing evidence based practice: a consensus approach. Qual Saf Health Care 2005, 14:26-33.
