# Supplementary figures and images for: Potential determinants of health-care professionals’ use of survivorship care plans: a qualitative study using the theoretical domains framework
Source: Implement Sci. 2014 Nov 15;9:167. doi: 10.1186/s13012-014-0167-z (PMC4236456; doi:10.1186/s13012-014-0167-z)

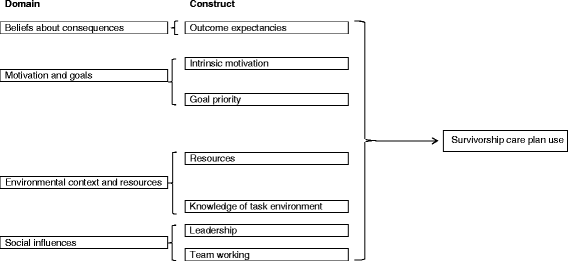

Supplement: Supplementary file 5 — Authors’ original file for figure 1 [file 13012_2014_167_MOESM5_ESM.gif]
