# Supplementary material for: Potential determinants of health-care professionals’ use of survivorship care plans: a qualitative study using the theoretical domains framework
Source: Implement Sci. 2014 Nov 15;9:167. doi: 10.1186/s13012-014-0167-z (PMC4236456; doi:10.1186/s13012-014-0167-z)
Supplement: Supplementary file 6 — Authors’ original file for figure 2 [file 13012_2014_167_MOESM6_ESM.docx]

**Table 1.** Judgment of theoretical domain relevance*

| **Specific belief by domain** | **Total frequency of mentions** | **High frequency?**** | **Conflicting beliefs present?†** | **Strength of beliefs‡** |
| --- | --- | --- | --- | --- |
|  |  |  |  |  |
| Knowledge |  |  |  |  |
| **Using SCPs is required.** | **9** | **yes** | **yes** | **moderate** |
| SCPs are a resource for survivors and their providers. | 11 | yes | no | weak |
|  |  |  |  |  |
| Skills |  |  |  |  |
| Training facilitates SCP use. | 3 | no | no | moderate |
| Using SCPs requires information technology skills. | 7 | no | no | weak |
| **Using SCPs requires clinical knowledge.** | **11** | **yes** | **no** | **strong** |
| Using SCPs requires management skills. | 2 | no | no | weak |
| Using SCPs requires communication skills. | 8 | yes | no | moderate |
| Using SCPs requires attention to detail. | 2 | no | no | moderate |
| Using SCPs requires a clinical degree. | 2 | no | yes | weak |
|  |  |  |  |  |
| Social/professional role and identity |  |  |  |  |
| **Using SCPs is compatible with my professional role.** | **13** | **yes** | **yes** | **moderate** |
|  |  |  |  |  |
| Beliefs about capabilities |  |  |  |  |
| SCPs need 'just the right amount' of information to be useful to providers. | 1 | no | no | moderate |
| SCP use depends on survivors' characteristics. | 10 | yes | no | weak |
| A large volume of survivors is challenging to using SCPs. | 5 | no | no | strong |
| I am confident in my ability to use SCPs. | 10 | yes | no | weak |
| **Gathering information is a barrier to using SCPs.** | **3** | **no** | **no** | **strong** |
|  |  |  |  |  |
| **Beliefs about consequences** |  |  |  |  |
| **The system as a whole benefits from SCP use.** | **13** | **yes** | **yes** | **moderate** |
| **Providers benefit from using SCPs.** | **11** | **yes** | **yes** | **moderate** |
| **Survivors benefit from SCPs.** | **13** | **yes** | **yes** | **strong** |
| Survivors respond well to SCPs. | 10 | yes | no | weak |
| SCPs transition survivors from cancer treatment. | 7 | no | yes | weak |
|  |  |  |  |  |
| **Motivation and goals** |  |  |  |  |
| **I advocate SCP use.** | **12** | **yes** | **no** | **strong** |
| **Using SCPs competes for my time.** | **12** | **yes** | **yes** | **strong** |
|  |  |  |  |  |
| Memory, attention, and decision processes |  |  |  |  |
| **A list helps me to use SCPs.** | **12** | **yes** | **yes** | **weak** |
| When I forget to use SCPs, I use an old method of transitioning survivors. | 2 | no | no | weak |
| I might not use SCPs if doing so were out of context | 4 | no | no | weak |
| I schedule SCP use when it is most effective/efficient for me. | 2 | no | no | weak |
|  |  |  |  |  |
| **Environmental context and resources** |  |  |  |  |
| **SCPs are delivered at a visit devoted to transitioning survivors.** | **5** | **no** | **yes** | **strong** |
| **A 'system' facilitates SCP use.** | **11** | **yes** | **yes** | **strong** |
| **Information technology supports SCP use.** | **12** | **yes** | **yes** | **strong** |
| **Funding facilitates SCP use.** | **9** | **yes** | **yes** | **strong** |
|  |  |  |  |  |
| **Social influences** |  |  |  |  |
| Survivor needs require SCP delivery to be as convenient as possible. | 8 | yes | no | moderate |
| **Using SCPs is an organizational priority.** | **4** | **no** | **yes** | **moderate** |
| **Influential people in the cancer program support SCP use.** | **12** | **yes** | **yes** | **weak** |
| External stakeholders support SCP use. | 6 | no | yes | weak |
| SCP use varies across the cancer program. | 6 | no | yes | weak |
| **Using SCPs requires buy-in from people who could assist in using SCPs.** | **13** | **yes** | **no** | **strong** |
|  |  |  |  |  |
| Emotion |  |  |  |  |
| It feels good to know that using SCPs helps survivors and their providers. | 8 | yes | no | moderate |
| Using SCPs helps me to feel calm when I transition survivors. | 3 | no | no | weak |
| Using SCPs is stressful. | 6 | no | no | moderate |
|  |  |  |  |  |
| Behavioral regulation |  |  |  |  |
| Feedback facilitates SCP use. | 11 | yes | no | weak |
| Using SCPs takes practice. | 6 | no | no | weak |
| I prepare to use SCPs by gathering information. | 12 | yes | no | weak |
| I 'sell' SCP use to promote buy-in from others. | 7 | no | yes | moderate |
| I use SCPs as a tool for communicating with survivors. | 12 | yes | no | weak |
| **I modify how I use SCPs to meet specific needs.** | **10** | **yes** | **yes** | **moderate** |
| I follow up with survivors to ensure that SCPs meet their needs. | 3 | no | no | weak |
|  |  |  |  |  |
| Nature of the behavior |  |  |  |  |
| Using SCPs is a 'work in progress.' | 6 | no | yes | moderate |
| Survivors are expected to deliver SCPs. | 1 | no | no | weak |
| SCPs should be kept up to date over time. | 3 | no | no | weak |
| SCPs present privacy concerns. | 1 | no | yes | weak |
| There are many ways SCP use can be initiated. | 4 | no | no | weak |
| **SCP use should begin during or prior to treatment.** | **5** | **no** | **yes** | **moderate** |

*Domains and specific beliefs judged to be relevant are in bold font.

**A relatively high frequency of specific beliefs (greater than the mean of 7 participants referring to the specific belief)

†Conflicting beliefs across participants

‡Evidence of strong beliefs (i.e., participants indicated that the specific belief contributed or would contribute to SCP use)
